# Proteomics and Machine Learning in the Prediction and Explanation of Low Pectoralis Muscle Area

**DOI:** 10.21203/rs.3.rs-3957125/v1

**Published:** 2024-03-04

**Authors:** Nicholas A. Enzer, Joe Chiles, Stefanie Mason, Toru Shirahata, Victor Castro, Elizabeth Regan, Bina Choi, Nancy F. Yuan, Alejandro A. Diaz, George R. Washko, Merry-Lynn McDonald, Raul San José Estépar, Samuel Y. Ash

**Affiliations:** Brigham and Women’s Hospital; University of Alabama at Birmingham; Brigham and Women’s Hospital; Brigham and Women’s Hospital; Boston University School of Medicine; National Jewish Health; Brigham and Women’s Hospital; University of California at San Diego; Brigham and Women’s Hospital; Brigham and Women’s Hospital; University of Alabama at Birmingham; Brigham and Women’s Hospital; Tufts University School of Medicine

## Abstract

Low muscle mass is associated with numerous adverse outcomes independent of other associated comorbid diseases. We aimed to predict and understand an individual’s risk for developing low muscle mass using proteomics and machine learning. We identified 8 biomarkers associated with low pectoralis muscle area (PMA). We built 3 random forest classification models that used either clinical measures, feature selected biomarkers, or both to predict development of low PMA. The area under the receiver operating characteristic curve for each model was: clinical-only = 0.646, biomarker-only = 0.740, and combined = 0.744. We displayed the heterogenetic nature of an individual’s risk for developing low PMA and identified 2 distinct subtypes of participants who developed low PMA. While additional validation is required, our methods for identifying and understanding individual and group risk for low muscle mass could be used to enable developments in the personalized prevention of low muscle mass.

## Introduction

Sarcopenia is a clinical syndrome characterized by low muscle strength and low muscle quality or quantity, and its presence is often associated with low physical performance.^1,2^ While sarcopenia often considered a result or a complication of age and comorbid conditions, sarcopenia as a disease in and of itself is independently associated with numerous adverse outcomes including injury, disease, and mortality.^1^ Thus it is crucial to identify those at risk for developing sarcopenia in order to intervene before adverse outcomes occur.^3^

One approach to measuring the low muscle quantity aspect of sarcopenia is the use of computed tomography (CT), including the measurement of pectoralis muscle area (PMA) on CT imaging of the chest. Prior work has demonstrated the utility of these measurements for predicting adverse outcomes such as exacerbations of respiratory disease and death.^4,56^ In addition, a variety of clinical factors and biomarkers have been identified as being associated with low muscle mass, such as comorbid conditions, demographics such as age, and biomarkers such as those associated with inflammation.^3,7–9^ However, little research has been conducted evaluating the prediction of incident low muscle mass, a key problem that must be addressed in order to help prevent it from occurring, and the studies that do exist are often limited by a small sample size or a lack of longitudinal data.^5,6,9^ Additionally, more work needs to be done examining what drives the risk for low muscle mass on the individual level. This is especially relevant as the benefits of precision-based approaches to medicine over disease-based approaches have become more realized in the medical community. Muscular dystrophies, sarcopenia, and cachexia have all been viewed as appropriate for undergoing precision-based care due to the variability of patients’ genetic makeup, health, and exposure to therapies.^11^

We leveraged longitudinal data collected from a large cohort of current and/or former smokers to identify peripheral protein blood biomarkers associated with the development of CT-derived PMA.^12^ In hopes of identifying those at highest risk for developing low PMA, we hypothesized that we could predict the development of low PMA by using a machine learning classification model that utilizes the identified biomarkers in conjunction with clinical measures and demographics. Additionally, we aimed to not only predict low muscle mass but also to illustrate and understand individual and group risk for it.

## Results

### Participant Characteristics

The Genetic Epidemiology of COPD (COPDGene) study enrolled 10,305 participants at baseline. For this study the analysis was limited to the 598 current and/or former smoking participants and 98 never-smoking control participants with complete data available *(e-Figure 1).* The current and/or former smoking cohort was made up of 48% men and 52% women. The cohort was 10.7% Black and 89.3% White. The mean age and BMI were 61.8 and 28.9 respectively. 36.3% were current smokers, 63.7% were former smokers, and the mean pack years was 42.9. Among the never-smoking control group, the 25th percentile of gender-stratified PMA at baseline was 44.9 cm^2^ for men (n = 32) and 24.5 cm^2^ for women (n = 66). Based on these values, there were 415 current and/or former smoking participants who did not have low PMA at baseline and 183 who did. Of the 415 current and/or former smoking participants that did not have low PMA at baseline, 22.9% developed low PMA at phase 2 (Table 1).

### Biomarker Feature Selection

There were 355 peripheral protein blood biomarkers that passed the univariate screen. Of those, 8 biomarkers were deemed important for predicting the development of low PMA by the Boruta feature selection algorithm: Histone acetyltransferase type B catalytic subunit (Hat1), Secreted protein acidic and rich in cysteine (SPARC), Lymphotoxin alpha 1/ beta 2 (Lymphotoxin a1/b2), Growth/differentiation factor 15 (GDF15), Cell adhesion molecule-related/down-regulated by oncogenes (CDON), Neurexophilin-1 (NXPH1), Vascular cell adhesion protein 1 (VCAM-1), and EGF-containing fibulin-like extracellular matrix protein 1 (EFEMP1) (Table 2).

### Predicting Low PMA with Machine Learning

**Figure 1:**
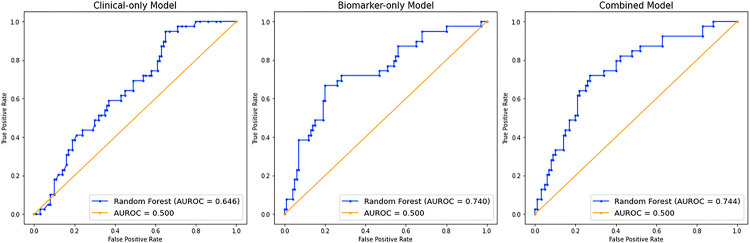
Predicting Low Pectoralis Muscle Area Areas under the receiver operating characteristic curves (AUROC) of our 3 random forest classification models built to predict low pectoralis muscle area (PMA). Five clinical measures were used in the clinical-only model: age, gender, pack years, height, and weight. Eight feature selected biomarkers for predicting the development of low PMA were used in the biomarker-only model: Histone acetyltransferase type B catalytic subunit (Hat1), Secreted protein acidic and rich in cysteine (SPARC), Lymphotoxin alpha 1/ beta 2 (Lymphotoxin a1/b2), Growth/differentiation factor 15 (GDF15), Cell adhesion molecule-related/down-regulated by oncogenes (CDON), Neurexophilin-1 (NXPH1), Vascular cell adhesion protein 1 (VCAM-1), and EGF-containing fibulin-like extracellular matrix protein 1 (EFEMP1). The combined model used predictors from both the clinical-only and biomarker-only models. The combined model and the clinical-only model were significantly different (P = 0.032). The combined model and the biomarker-only model were not significantly different (P = 0.78). The clinical-only model and the biomarker-only model were not significantly different (P = 0.09).

Regarding the prediction models’ discrimination (Fig. 1), the clinical-only model had an area under the receiver operating characteristic curve (AUROC) of 0.646, which was worse than the biomarker-only model’s AUROC of 0.740, but their difference did not reach statistical significance (p for AUC comparison = 0.093). The combined model had better discrimination than the clinical-only model with an AUROC of 0.744 (p for comparison = 0.032) but was not better than the biomarker-only model (p for comparison = 0.779). Model calibration curves are found in the supplementary material. The Brier scores of the combined model and the biomarker-only model were identical (0.174) while the Brier score of the clinical-only model was slightly higher (0.203). (Combined model: *e-Figure 2,* clinical-only and biomarker-only models: *e-Figures 3–4).* The testing set included 139 participants and the training set included 168 participants after down sampling (276 originally).

### Individual Risk

**Figure 2:**
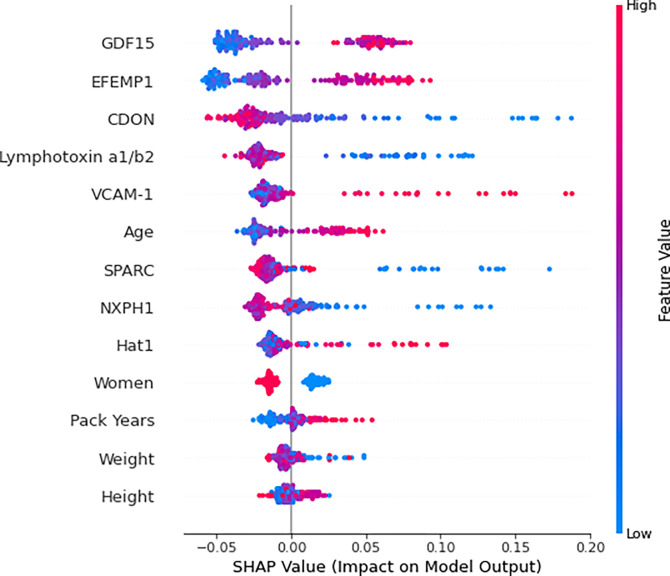
Combined Model Summary Plot The combined random forest classification model’s training set’s (n = 168) predictors ordered by importance for predicting low pectoralis muscle area (PMA). Shapley additive explanation (SHAP) values indicate the predictors’ impact on the probability of developing low PMA. For numeric predictors, red indicates a high value and blue indicates a low value. For the sole categorical predictor, “Women”, red and blue represent women and men respectively. Five clinical measures were used: age, gender, pack years, height, and weight. Eight feature selected biomarkers for predicting the development of low PMA were used: Histone acetyltransferase type B catalytic subunit (Hat1), Secreted protein acidic and rich in cysteine (SPARC), Lymphotoxin alpha 1/ beta 2 (Lymphotoxin a1/b2), Growth/differentiation factor 15 (GDF15), Cell adhesion molecule-related/down-regulated by oncogenes (CDON), Neurexophilin-1 (NXPH1), Vascular cell adhesion protein 1 (VCAM-1), and EGF-containing fibulin-like extracellular matrix protein 1 (EFEMP1).

For the combined model, the order of importance of the predictors was GDF15, EFEMP1, CDON, Lymphotoxin a1/b2, VCAM-1, age, ON, NXPH1, Hat1, gender, pack years, height, and weight (Fig. 2). Feature importance analysis of the clinical-only and biomarker-only models are found in the supplementary material *(e-Figures 5–6).*

**Figure 3:**
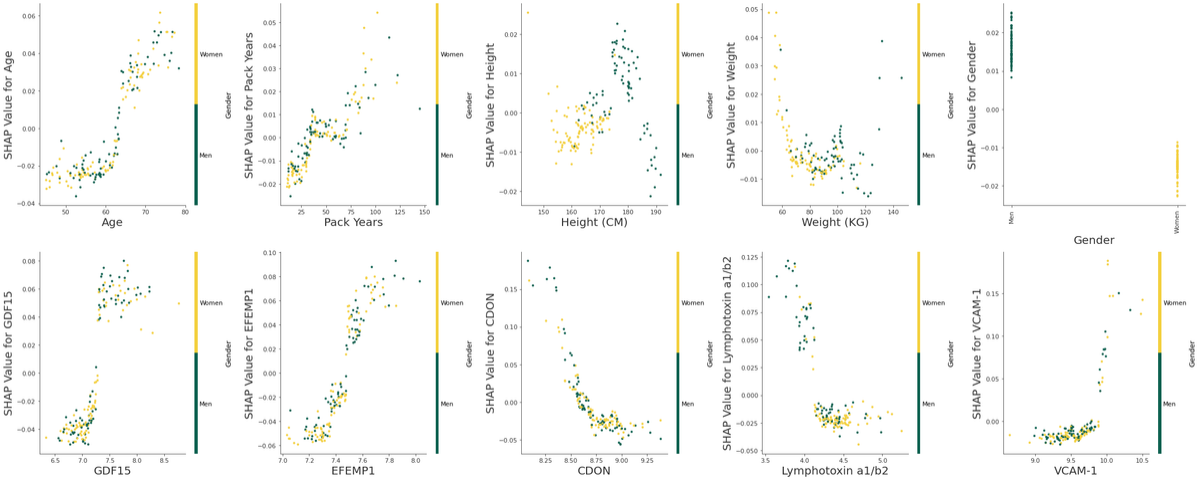
Predictor Measurements vs. Shapley Additive Explanation Values (Combined Model) The relationships between the clinical predictors: age, pack years, height, weight, and gender, and the 5 most important feature selected biomarkers for predicting the development of low pectoralis muscle area (PMA): Growth/differentiation factor 15 (GDF15), EGF-containing fibulin-like extracellular matrix protein 1 (EFEMP1), Cell adhesion molecule-related/down-regulated by oncogenes (CDON), Lymphotoxin alpha 1/beta 2 (Lymphotoxin a1/b2), Vascular cell adhesion protein 1 (VCAM-1) with their respective Shapley additive explanation (SHAP) values. SHAP values indicate the predictors’ impact on the probability of developing low PMA. Yellow and green indicate whether the participant is a woman or a man respectively. This is solely examining the combined random forest classification model’s training set (n = 168).

**Figure 4:**
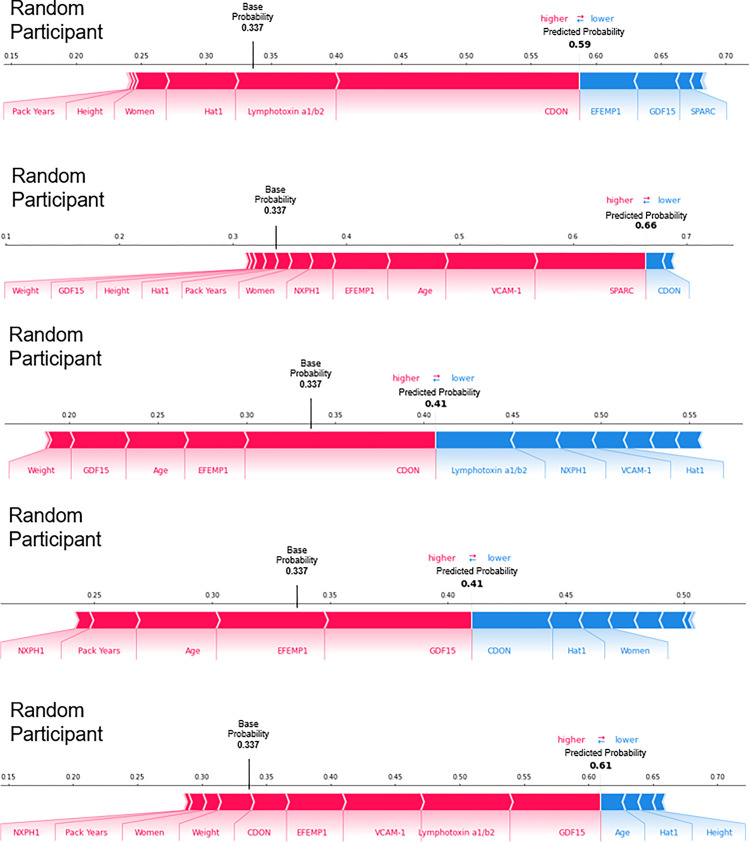
Force Plots for Participants with a Predicted Probability of Developing Low Pectoralis Muscle Area Greater than the Mean Probability of the Combined Model’s Training Set Force plots for 5 randomly selected participants from the combined random forest classification model’s training set (n = 168) with a predicted probability of developing low pectoralis muscle area (PMA) greater than the mean probability of the combined model’s training set (0.337). Each predictor has a Shapley additive explanation (SHAP) value that indicates the predictors’ impact on the probability of developing low PMA. Red and blue indicate whether the impact is positive or negative respectively. Five clinical measures were used: age, gender, pack years, weight, and height. Eight feature selected biomarkers for predicting the development of low PMA were used: Histone acetyltransferase type B catalytic subunit (Hat1), Secreted protein acidic and rich in cysteine (SPARC), Lymphotoxin alpha 1/ beta 2 (Lymphotoxin a1/b2), Growth/differentiation factor 15 (GDF15), Cell adhesion molecule-related/down-regulated by oncogenes (CDON), Neurexophilin-1 (NXPH1), Vascular cell adhesion protein 1 (VCAM-1), and EGF-containing fibulin-like extracellular matrix protein 1 (EFEMP1).

Visual evaluation of the relationships between the measurements of each model’s training set’s (n = 168) predictors and their respective Shapley additive explanation (SHAP) values suggests that several may have definable thresholds. For example, for the combined model, GDF15 and EFEMP1 had breakpoints near the middle of their range. (Combined model: Fig. 3, *e-Figure 7,* clinical-only and biomarker-only models: *e-Figures 8–9.)* In addition, visual evaluation of the force plots from 10 randomly selected participants revealed a large amount of heterogeneity in the covariates that drive the individual participant’s final predicted probability. The mean predicted probability of the combined, biomarker-only, and clinical only models’ training sets were 0.337, 0.337, and 0.333 respectively (combined model: Fig. 4, *e-Figure 10,* clinical-only and biomarker-only models: *e-Figures 11–14).*

### Group Risk

**Figure 5:**
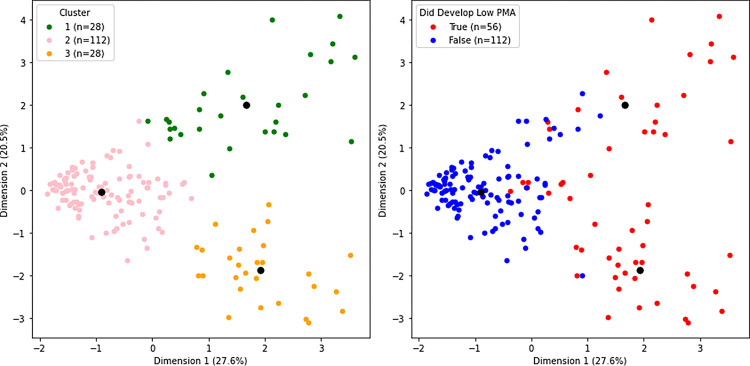
Clustering Participants via Principal Component Analysis and K-Means Clustering The plot on the left illustrates the participants in the training set (n = 168) of the combined random forest classification model, for predicting the development of low PMA, clustered based on the similarity of their feature selected biomarkers’ Shapley additive explanation (SHAP) values using principal component analysis (PCA) and K-Means clustering. There were 2 PCA components. The plot on the right illustrates whether the individuals in the clusters did or did not develop low pectoralis muscle area (PMA). Black dots indicate the centroids of the clusters. The SHAP values of eight feature selected biomarkers for predicting the development of low PMA were used: Histone acetyltransferase type B catalytic subunit (Hat1), Secreted protein acidic and rich in cysteine (SPARC), Lymphotoxin alpha 1/ beta 2 (Lymphotoxin a1/b2), Growth/differentiation factor 15 (GDF15), Cell adhesion molecule-related/down-regulated by oncogenes (CDON), Neurexophilin-1 (NXPH1), Vascular cell adhesion protein 1 (VCAM-1), and EGF-containing fibulin-like extracellular matrix protein 1 (EFEMP1).

**Figure 6:**
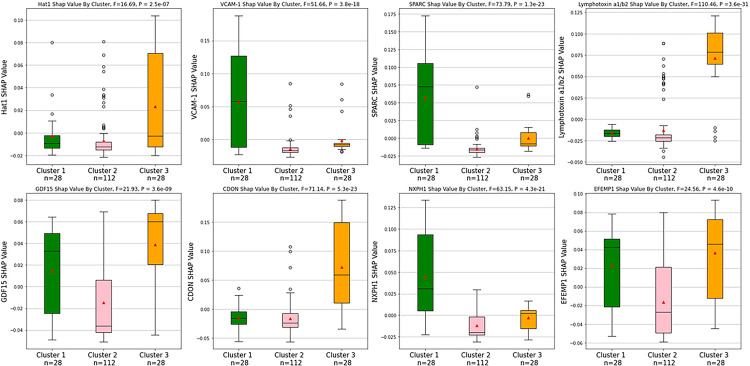
Comparing Feature Selected Biomarker Shapley Additive Explanation Values between Clusters Box plots comparing the feature selected biomarkers for predicting the development of low PMA’s SHAP values between the 3 clusters that were illustrated using principal component analysis (PCA) and K-Means clustering. All the biomarkers’ SHAP values were significantly different between the 3 clusters via one-way ANOVA (P < 0.001). Eight feature selected biomarkers for predicting the development of low PMA were used: Histone acetyltransferase type B catalytic subunit (Hat1), Secreted protein acidic and rich in cysteine (SPARC), Lymphotoxin alpha 1/ beta 2 (Lymphotoxin a1/b2), Growth/differentiation factor 15 (GDF15), Cell adhesion molecule-related/down-regulated by oncogenes (CDON), Neurexophilin-1 (NXPH1), Vascular cell adhesion protein 1 (VCAM-1), and EGF-containing fibulin-like extracellular matrix protein 1 (EFEMP1). The black lines indicate the medians, the red triangles indicate the means, the circles represent outliers, and the error bars represent 1.5x the interquartile range. There were 168 participants between the 3 groups.

K-Means clustering resulted in 3 distinct clusters of participants based on the silhouette coefficient. Performing principal component analysis (PCA) on the combined model’s biomarkers’ standardized SHAP values resulted in the first component explaining 27.6% of the variance and the second component explaining 20.5% of the variance. When stratified for the development of low PMA, one cluster was predominantly made up of participants who did not develop low PMA, and the remaining 2 clusters were predominantly made up of participants who did develop low PMA (Fig. 5). All the feature selected biomarkers’ SHAP values were significantly different between the 3 clusters via one-way ANOVA (P< 0.001). The clusters that were predominantly made up of participants who developed low PMA had different SHAP profiles from one another despite having the same outcome. The cluster that was predominantly made up of participants who did not develop low PMA had consistently low SHAP values (Fig. 6).

### Feature Selected Biomarkers Relationship with PMA

Finally, of the 5 most important feature selected biomarkers, baseline EFEMP1 was significantly (P = 0.008) negatively correlated (r = −1.29) with PMA change. Baseline CDON was significantly (P = 0.009) positively correlated (r = 0.127) with PMA change. The remaining 3 biomarkers at baseline were not significantly correlated with PMA change (Table 3).

## Discussion

Leveraging longitudinal data from the COPDGene study, we developed a machine learning classification model that predicted the development of low PMA in smokers using clinical measures, demographics, and peripheral protein blood biomarkers. This model outperformed a model that utilized only clinical measures and demographics as predictors and performed similarly to one that incorporated biomarker information only. In addition, subsequent analysis of the models suggests that there may be specific cut-points of interest for the biomarkers identified, and that there is a large amount of heterogeneity in what drives an individual patient’s risk for developing low PMA. This heterogeneity was used to cluster the participants into distinct subtypes.

This work has several strengths, one of which is the use of a large-scale longitudinal research cohort that enabled the prediction of low muscle mass utilizing an abundance of protein biomarkers in the initial panel. Prior efforts to predict low muscle mass using biomarkers have often been cross-sectional with relatively small and non-diverse cohorts and with relatively small candidate biomarker panels.^7,10,13^ Also, by utilizing all-relevant feature selection tools such as Boruta, we were able to select a small number of relevant biomarkers of interest. Subsequent evaluation using SHAP analysis and K-Means clustering provided insights into potential threshold values for those biomarkers as well as demonstrating the heterogeneity in what contributes to a specific individual’s probability of developing low PMA. We believe our methods for biomarker selection and analyzing patient risk are novel to the issue of low muscle mass.

In terms of specific findings, the 8 biomarkers that were deemed important for predicting low PMA were surprisingly diverse, with roles ranging from leukocyte migration regulation to histone acetylation.^14,15^ Some of the biomarkers found validated prior research. For example, serum GDF15 has been identified as a potential biomarker for sarcopenia due to it being negatively correlated with muscle mass^16^ and muscle power^17^ in humans. Although, we could not find any research relating circulating CDON to muscle mass, it has been shown that mice with satellite cell-specific CDON ablation had impaired muscle generation^18^ and it is believed that CDON positively regulates skeletal myogenesis.^19,20^

Interestingly, some of the biomarkers found contradicted prior research. For example, Hat1-haplodeficient mice have been revealed to have a shorter lifespan and more premature age-related phenotypes, including muscle atrophy, than wildtype mice.^21^ Moreover, satellite cell VCAM-1 null mice had delayed, or decreased myofibril growth compared to wildtype mice.^22^ These contradictions may be due to species differences and contrasts in function between circulating biomarkers and biomarkers’ expression in muscle, a notable weakness of our current work which relies on peripheral biomarkers.

Some of the biomarkers found may help elucidate prior unclear research. For example, a cross-species meta-analysis identified EFEMP1 as consistently overexpressed in the muscle with age, and even consistently overexpressed in all studied tissues in their analyses.^23^ However, there are areas where EFEMP1 appears to be reduced during aging such as the superficial zone of the articular cartilage^24^, and mice with inactivated EFEMP1 appear to age prematurely.^25^ In our study, EFEMP1 was found to increase the likelihood of developing low PMA in our model, and it was found that EFEMP1 measurements were higher in the cohort that had low PMA at baseline. Altogether, this suggests that the upregulation of EFEMP1 may be an adaptive response to delay the inevitable aging and muscle loss processes. Similarly, conflicting data also exists for the role of SPARC in muscle biology and sarcopenia. For example, there has been evidence that SPARC both positively and negatively effects the differentiation of myoblasts.^26,27^ Moreover, one group found that serum SPARC was significantly higher in a sarcopenic cohort compared to a non-sarcopenic cohort while, another group found the opposite, although the latter finding was not statistically significant and there were concurrent disease processes.^7,28^ In our study SPARC was found to decrease the likelihood of developing low PMA in our model, and it was found that SPARC measurements were higher in the cohort that did not have low PMA at baseline. Together, this suggests that SPARC likely has a negative role in the complex muscle loss process. Hopefully, our results concerning EFEMP1 and SPARC will help minimize the ambiguity of these biomarkers.

With regards to the identification of novel biomarkers related to low muscle mass, neither NXPH1 nor Lymphotoxin a1/b2 appear to have a connection with low muscle mass in the literature. Whether our findings reflect true associations or confounding is unclear and further work is needed to better elucidate what roles, if any, these proteins may play in the development of low muscle mass.

Interestingly, when assessing the feature importance of the combined model’s predictors we noticed that the protein biomarkers appeared more important than most of the clinical predictors. While this could be taken to support the use of proteomics for identifying those at risk for low muscle mass, it is important to caution that there are numerous other clinical predictors that can and should be evaluated, including both complicated screening tools as well as simple clinical questions related to weight loss and exercise capacity. These extensive analyses are beyond the scope of this current investigation but should be done to better explore these issues.

Notably, for the quantitative predictors in our models there is a greater range of positive impact values than negative impact values. In other words, the models avoid giving strong negative impact values regardless of the predictors’ actual values, insinuating that there is not one realistic predictor value that can drastically negatively affect the model’s outcome. Interestingly, the 5 most important biomarkers for predicting low PMA, when assessed individually at baseline, were not highly correlated with change in PMA between baseline and phase 2. This highlights the potential strength of tools such as machine learning to identify predictors that may not be readily apparent when using more traditional statistical analyses. Similarly, tools such as SHAP analysis may enable insights into specific relationships between predictors and outcomes. For example, plotting the SHAP values against the predictor measurements allowed us to examine the threshold at which the impact direction changes. The plots for age and pack years are especially illustrative. This information may help determine threshold values for concern in clinical applications. The SHAP force plots also help illustrate what is happening on the individual level and show the multifactorial nature of low muscle mass. This could be especially helpful when considering personalized medicine approaches to specific patients, as different patients may have different pathobiological processes responsible for the same phenotype, and thus they may respond differently to targeted therapy. Our cluster analysis supports this theory as they illustrated 2 distinct subtypes of participants who developed low PMA. This could be due to differences in biomarker profiles, or perhaps due to underlying conditions, for example, aging and smoking-related disease. Interestingly, of the 3 clusters, it appears that the cluster that mostly did not develop low PMA is the densest cluster, and therefore has a less variance than the other 2 clusters. Perhaps this consistency is indicative of a “normal” profile subtype. As expected, when comparing the biomarkers’ SHAP profiles between the 3 clusters, the cluster that was mostly composed of those who did not develop low PMA consistently had the lowest SHAP values (when examining the median). The other 2 clusters had considerably different biomarker SHAP profiles from one another. For example, the participants in Cluster 1 developed low PMA with CDON and Lymphotoxin a1/b2 having a negative impact on their predicted probability for developing low PMA. On the other hand, Cluster 3 developed low PMA with CDON and Lymphotoxin a1/b2 having a positive impact on their predicted probability for developing low PMA. Surprisingly, the most important biomarkers overall, GDF15 and EFEMP1, had similar SHAP values in both clusters, indicating that it may be the less important biomarkers that are the most responsible for this stratification.

Clinically, this study demonstrates that it may be possible to identify patients at highest risk for low muscle mass before it develops, potentially enabling targeted interventions ranging from diet and exercise to current and novel pharmacologic therapies. This is especially important given both the growing recognition of the benefits of personalized medicine and the growing recognition that muscle loss, while often related to other co-morbid diseases, is a distinct process independently associated with morbidity and mortality. Finally, our approach to biomarker selection and risk analysis is not unique to low muscle mass and could be expanded to other domains as well, potentially enabling the identification of important biomarkers and underlying pathways for other clinical problems.

Unfortunately, this project had several limitations. We did not have a validation cohort and the participants enrolled in this study were less diverse than the general population, which may reduce its generalizability. In addition, there is likely collinearity between some of the biomarkers and clinical measures. For example, plasma GDF15 has been shown to be significantly positively associated with age.^29^ It is therefore difficult to separate the effects of age from the effects of specific protein biomarkers. Moreover, SHAP analyses assume independence between the predictors, which may not be the case. Finally, although the feature importance results are interesting, they do not indicate causality, only association, significantly limiting their interpretation.

In summary, using proteomics and machine learning, we identified protein biomarkers associated with low PMA in smokers, developed risk prediction tools able to predict the development of low PMA over 5 years of follow-up, and analyzed individual risk and group risk for developing low PMA.

## Methods

### Parent Study

Data was acquired through COPDGene study: an ongoing longitudinal observational study that examines the development of chronic obstructive pulmonary disease in smokers. There were 10,198 current and/or former smokers and 107 non-smoking control participants initially enrolled in COPDGene (*e-Figure 1*). All participants were non-Hispanic white or African American, and all current and/or former smokers had a minimum of 10 pack years. Data was collected at baseline (phase 1) and after 5 years of follow-up (phase 2). Additional phase 3, 10-year follow up visits are currently in progress and are not included in this current study. Data used for this study included an extensive questionnaire at baseline, CT of the chest at baseline and phase 2, and peripheral protein blood biomarker measurements via the SomaScan assay at baseline. The biomarkers were measured in relative fluorescent units and the measurements were normalized and natural log transformed.^30^ PMA (cm^2^) was derived using a single axial CT image at the level of the aortic arch and the suprasternal notch using a previously described method.^5^ All research was performed in accordance with relevant guidelines. All participants provided written informed consent, and the study was approved by the institutional review board at each of the 21 centers including Brigham and Women’s Hospital.^12^

### Defining Low PMA

For this study, we defined the current and/or former smokers as having low PMA if they had a PMA that was less than the 25th percentile of baseline never-smoking control participants, stratified by gender. We defined the current and/or former smokers as having low PMA at baseline and at phase 2.

### Biomarker Feature Selection

To identify protein biomarkers of interest, we performed an initial univariate screen comparing mean biomarker measurements in current and/or former smokers with (n = 183) and without (n = 415) low PMA at baseline. There were 1,317 initial biomarkers and only the biomarkers with a Welch’s t-test false discovery rate (FDR) q < 0.10 were retained. We then utilized Boruta feature selection with a one-step correction to identify the most relevant biomarkers for predicting the development of low PMA, i.e., the change from not having low PMA at baseline to having low PMA at the 5-year follow-up visit. The default parameters were used except for the number of estimators which was set to ‘auto’ and the maximum depth which was set to 8. Boruta was chosen due to it being an all-relevant feature selection method, meaning that it aims to uncover all the relevant features as opposed to uncovering the minimal number of features that score well.^31,32^

### Predicting Low PMA with Machine Learning

To identify participants at highest risk for developing low PMA and to determine the utility of clinical and biomarker data to predict low PMA, we built 3 random forest classification models to predict the development of low PMA, i.e. the change from not having low PMA at baseline to having low PMA at the 5-year follow-up visit.^33^ The first was a clinical-only model that incorporated easily attainable baseline clinical measures (height, weight, pack years) and demographics (age and gender). The second was a biomarker-only model that incorporated the baseline protein biomarkers selected using the feature selection process. The third model incorporated both the clinical measures/demographics and the selected biomarkers. All models were trained on the same 2/3 random sample and tested on the remaining 1/3. Finally, 2:1 down-sampling was performed to account for event prevalence. Model hyperparameters were tuned using Bayesian optimization. The models’ performances were summarized by the AUROC, the calibration curve, and the Brier score (“the mean squared difference between the predicted probability and the actual outcome”) of their respective testing sets.^33^ The calibration curves were calculated using 10 bins.

### Individual Risk

To assess the importance of the combined model’s individual predictors and to examine the predictors’ impact (strength and direction) on the predicted probability for developing low PMA, a SHAP summary plot was built.^34^ SHAP plots utilize SHAP values which are assigned to each predictor and indicate how much the predictor, alone, contributes to a model’s prediction. This is based on the game theory idea of Shapley values which represent the average marginal contribution of a predictor across all possible combinations of predictors. In other words, on the individual level, the difference between the predicted probability and the expected (base) probability is the sum of the SHAP values for every predictor.^34,35^ To determine if there were possible threshold values for the predictors, the clinical measurements and the 5 most important biomarker measurements were then plotted against their respective SHAP values. In addition, to visualize how SHAP values were affecting the prediction on the individual level, SHAP force plots were built for 10 randomly selected individuals: 5 predicted to develop low PMA and 5 predicted to not develop low PMA (using the mean predicted probability of the combined model’s training set as the cutoff point).^36^ All SHAP analyses focused on the training set of the combined model unless otherwise specified.

### Group Risk

Additionally, to examine whether there were any distinguishable groups within the participants, we clustered the combined model’s training set based on the biomarkers’ standardized SHAP values. This was done using PCA, to reduce dimensionality, and K-Means clustering. The optimal number of clusters was based on the silhouette coefficient of the raw SHAP values. We then stratified the clusters based on whether the participants developed low PMA in phase 2. Differences in the biomarkers’ raw SHAP values between the 3 clusters were then assessed using a one-way ANOVA and visualized using box plots. All SHAP analyses focused on the training set of the combined model unless otherwise specified.

### Feature Selected Biomarkers Relationship with PMA

Finally, to explore the relevance of the 5 most important biomarkers, Pearson correlation coefficients were calculated between the biomarkers at baseline and the change in PMA between the 2 phases (cm^2^) amongst participants without low PMA at baseline.

### Statistics

All analyses were conducted using Python 3.9.7 and R 4.0.3. All statistical tests were 2-tailed and P values < 0.05 were taken to mean statistical significance unless otherwise specified. The initial univariate screen included a Welch’s t-test where FDR q < 0.10 (calculated using the Benjamini-Hochberg procedure) was taken to mean statistical significance. The AUROCs were compared using a t-test. A one-way ANOVA and boxplots were used to examine and visualize the differences in biomarker SHAP values between clusters. Boxplots included means (red triangles), medians (black lines) and error bars (1.5x the interquartile range). Pearson correlation coefficients were calculated to examine the relationship between biomarkers and change in PMA between baseline and phase 2.

## Figures and Tables

**Table 1:** Baseline characteristics of COPDGene participants used in this study, non-stratified and stratified by low pectoralis muscle area at baseline.

		Baseline Characteristics	Low PMA at Baseline	No Low PMA at Baseline
n		598	183	415
Gender, n (%)	Men	287 (48.0)	117 (63.9)	170 (41.0)
	Women	311 (52.0)	66 (36.1)	245 (59.0)
Race, n (%)	Black	64 (10.7)	1 (0.5)	63 (15.2)
	White	534 (89.3)	182 (99.5)	352 (84.8)
Age, mean (SD)		61.8 (8.7)	66.7 (7.8)	59.7 (8.2)
BMI, mean (SD)		28.9 (5.7)	28.1 (5.8)	29.2 (5.6)
Smoking Status, n (%)	Current Smoker	217 (36.3)	53 (29.0)	164 (39.5)
	Former Smoker	381 (63.7)	130 (71.0)	251 (60.5)
Pack Years, mean (SD)		42.9 (23.6)	47.1 (24.7)	41.1 (22.9)
Developed Low PMA at Phase 2, n (%)		-	-	95 (22.9)

BMI = body mass index, COPDGene = Genetic Epidemiology of COPD, PMA = pectoralis muscle area, SD = standard deviation

**Table 2:** Biomarkers that underwent a univariate screen (Weltch’s t-Test, FDR q < 0.10) between those without and with low PMA at baseline and were considered relevant for predicting the development of low pectoralis muscle area via Boruta feature selection.

Biomarker	Mean (SD) No Low PMA at Baseline (n = 415)	Mean (SD) Low PMA at Baseline (n = 183)	T-statistic (P value)	FDR q value	Brief Description
Histone acetyltransferase type B catalytic subunit (Hat1)	6.04 (0.39)	5.96 (0.35)	2.66 (0.008)	0.038	Enzyme associated with the acetylation of newly synthesized histone H4.^15^
Vascular cell adhesion protein 1 (VCAM-1)	9.54 (0.27)	9.60 (0.26)	−2.60 (0.010)	0.045	Cell adhesion molecule whose expression is induced on endothelial cells during inflammatory disease. Plays a role in the regulation of leukocyte migration.^14^
Secreted protein acidic and rich in cysteine (SPARC)	9.99 (0.64)	9.75 (0.69)	4.13 (< 0.001)	< 0.001	Glycoprotein associated with the binding of cells and matrix components.^37^
Lymphotoxin alpha 1/beta 2 (Lymphotoxin a1/b2)	4.34 (0.27)	4.28 (0.30)	2.39 (0.017)	0.072	Cytokines associated with the adaptive immune response and the maintenance of lymphoid organ architecture.^38^
Growth/differentiation factor 15 (GDF15)	7.19 (0.39)	7.39 (0.37)	−6.00 (< 0.001)	< 0.001	Cytokine released in response to stress and tissue injury.^39^
Cell adhesion molecule-related/down-regulated by oncogenes (CDON)	8.76 (0.22)	8.68 (0.21)	3.94 (< 0.001)	0.001	Transmembrane glycoprotein associated with Hedgehog proteins and myoblast differentiation.^19,40^
Neurexophilin-1 (NXPH1)	8.93 (0.39)	8.83 (0.41)	2.84 (0.005)	0.025	Glycoprotein whose detected expression (in humans) is strongest in the spleen.^41^
EGF-containing fibulin-like extracellular matrix protein 1 (EFEMP1)	7.42 (0.18)	7.49 (0.21)	−3.90 (< 0.001)	0.001	Glycoprotein that has a role in basement membranes.^42^

Abbreviations: FDR = false discovery rate, PMA = pectoralis muscle area, SD = standard deviation

**Table 3:** Relationships between the 5 most important feature selected biomarkers at baseline for predicting low pectoralis muscle area and the change in pectoralis muscle area (cm^2) between baseline and phase 2 (n = 415).

Biomarker	Pearson Correlation Coefficient	P value
Growth/differentiation factor 15 (GDF15)	−0.049	0.317
EGF-containing fibulin-like extracellular matrix protein 1 (EFEMP1)	−0.129	0.008
Cell adhesion molecule-related/down-regulated by oncogenes (CDON)	0.127	0.009
Lymphotoxin alpha 1/ beta 2 (Lymphotoxin a1/b2)	0.048	0.332
Vascular cell adhesion protein 1 (VCAM-1)	−0.011	0.823

## Data Availability

The data that support the findings of this study are available from the database of Genotypes and Phenotypes (dbGaP https://www.ncbi.nlm.nih.gov/gap/, accession number pht002239.v4.p2), the National Heart, Lung and Blood Institute (NHLBI) BioData Catalyst (https://biodatacatalyst.nhlbi.nih.gov/resources/data), and by reasonable request from the COPDGene study (https://www.copdgene.org/).
